# Changes in Fecal Short-Chain Fatty Acids in IBS Patients and Effects of Different Interventions: A Systematic Review and Meta-Analysis

**DOI:** 10.3390/nu16111727

**Published:** 2024-05-31

**Authors:** Xuan Ju, Zhenliang Jiang, Jiayin Ma, Dong Yang

**Affiliations:** Beijing Key Laboratory of Functional Food from Plant Resources, College of Food Science & Nutritional Engineering, China Agricultural University, Beijing 100083, China; s20193060968@cau.edu.cn (X.J.); s20233061206@cau.edu.cn (Z.J.); s20223061123@cau.edu.cn (J.M.)

**Keywords:** irritable bowel syndrome, short-chain fatty acids, gut microbiota, low-FODMAP diet, FMT

## Abstract

Context: Short-chain fatty acids (SCFAs) have been reported to be associated with the pathogenesis of irritable bowel syndrome (IBS), but the results are conflicting. Objective: Here, a systematic review of case–control studies detecting fecal SCFAs in IBS patients compared with healthy controls (HCs) and self-controlled studies or randomized controlled trials (RCTs) investigating fecal SCFA alterations after interventions were identified from several databases. Data sources: A systematic search of databases (PubMed, Web of Science, and Embase) identified 21 studies published before 24 February 2023. Data extractions: Three independent reviewers completed the relevant data extraction. Data analysis: It was found that the fecal propionate concentration in IBS patients was significantly higher than that in HCs, while the acetate proportion was significantly lower. Low-FODMAP diets significantly reduced the fecal propionate concentration in the IBS patients while fecal microbiota transplantation and probiotic administration did not significantly change the fecal propionate concentration or acetate proportion. Conclusions: The results suggested that the fecal propionate concentration and acetate proportion could be used as biomarkers for IBS diagnosis. A low-FODMAP diet intervention could potentially serve as a treatment for IBS while FMT and probiotic administration need more robust trials.

## 1. Introduction

Irritable bowel syndrome (IBS) is one of the most common chronic gastrointestinal disorders, which substantially reduces the quality of life and work productivity [1]. It is subtyped into diarrhea-predominant IBS (IBS-D), constipation-predominant IBS (IBS-C), IBS with mixed bowel habits (IBS-M), and un-subtyped IBS (IBS-U) depending on the bowel habit abnormality [2]. However, the current understanding of IBS etiology and pathogenesis remains limited [3]. Its proposed pathogenesis mechanisms include altered pain perception, altered brain–gut interaction, dysbiosis, increased intestinal permeability, increased gut mucosal immune activation, and visceral hypersensitivity [4]. Recently, accumulating evidence has highlighted the role of the gut microbiota in the development of IBS [5,6], and numerous studies have reported differences in the gut microbiota of IBS patients compared with healthy controls (HCs) [7,8,9]. IBS exhibits a definite overall microbial signature, which produces a clear differentiation from healthy controls (HCs), and the composition varies depending on the predominant form of IBS [10]. Short-chain fatty acids (SCFAs), particularly acetate, propionate, and butyrate, not only exhibit important intestinal and immune-modulatory functions but also reflect the status of the intestinal flora [11,12,13].

Up to now, no specific and reproducible hallmarks have been identified and an IBS diagnosis, in some cases, may lead to a differential diagnosis problem, as suggested by a recent study [14]. The correlation between intestinal physiological characteristics and functional bowel disorders has been extensively investigated. Many studies have focused on the association between fecal SCFAs and IBS, whereas the conclusions are inconsistent or even contrary. For instance, Tana et al. showed that IBS patients possess significantly higher levels of total SCFAs including acetate and propionate than HCs, while Treem et al. proved that IBS-D patients had less total SCFAs and a lower acetate proportion than HCs; Tian et al. found that there was no significant difference between IBS patients and HCs with regard to the concentrations of fecal acetate, propionate, and butyrate [15,16,17]. To comprehensively understand these differences, we perform a meta-analysis to discover the potential association between the fecal SCFA concentration and IBS status from all previous studies, and explore whether fecal SCFAs can be utilized as biomarkers of an IBS diagnosis.

Restricting food with highly fermentable oligo-, di-, monosaccharides, and polyols (FODMAPs), is a novel treatment option [18]. Examples of foods containing FODMAPs include fruits (apple, pears, peaches, and watermelons), vegetables (onions, garlic, squash, and mushrooms), dairy products, grains (wheat and rye), and sweeteners (sorbitol and mannitol), etc. [19]. The low-FODMAP diet is effective in reducing the global symptoms and improving the bowel habits of adult IBS patients [20]. The modulation of the gut microbiota with agents such as probiotics, prebiotics, symbiotics, luminal antibiotics, and fecal microbiota transplantation (FMT) have also been suggested as treatment options for IBS, while a recent systematic review and meta-analysis did not support FMT as a successful treatment strategy in IBS [21]. Here, we also evaluate fecal SCFA alterations in IBS patients after receiving different interventions to provide a reference for the clinical treatment of IBS.

## 2. Materials and Methods

### 2.1. Protocol Registration and Reporting Format

The protocol of the present review was registered and allocated the identification number CRD42023452054 in the PROSPERO database, hosted by the National Institute for Health Research, University of York, Center for Reviews and Dissemination. This manuscript was prepared following the Cochrane Collaboration guidelines and is reported in accordance with the Preferred Reporting Items for Systematic reviews and Meta-Analysis Extension (PRISMA).

### 2.2. Information Sources and Search Strategy

A systematic literature search of online databases including PubMed, Web of Science, and Embase was performed for all published articles from inception to February 2023. The search terms included “irritable bowel syndrome”, “IBS”, “short-chain fatty acids”, “volatile fatty acids”, “SCFAs”, “acetate”, “propionate”, “butyrate”, and “valerate”. Boolean operators (AND, OR, NOT) were used to widen and narrow the results. Review articles and meeting abstracts were excluded during searching process as well as articles not in English or performed with animals (see Supplementary Methods, full electronic search strategy for each database including all limits and filters used). After screening the titles and abstracts, cited and citing reference searches were conducted in selected articles. The Systematic Review Registration: PROSPERO registration no CRD42023452054.

### 2.3. Study Selection and Eligibility Criteria

Titles and abstracts of retrieved records were investigated for relevance to our topic, then full texts of all potentially relevant articles were evaluated in detail according to the following inclusion and exclusion criteria. The inclusion criteria include the following: (1) the diagnosis of IBS was based on Rome criteria (issued at that time, or described as IBS before the issue of Rome criteria I in 1990); (2) the study design for comparing IBS patients with HCs was case–control, and for comparing treatment with pre-treatment or placebo was self-controlled or a randomized controlled trial (RCT); (3) available fecal SCFA data were sufficient to calculate mean difference (MD) or standardized mean difference (SMD) with 95% confidence interval (CI); (4) adult IBS patients and HCs were similar in age and sex. The exclusion criteria include the following: (1) studies in which IBS patients also suffered from other gastrointestinal diseases; (2) studies of pediatric patients or animals; (3) studies with no available full text or sufficient data for calculation of MD or SMD. Three reviewers (Xuan Ju, Zhenliang Jiang, and Jiayin Ma) evaluated each record that met the predetermined criteria independently, and any disagreement was resolved through mutual consultation or discussion with a fourth independent reviewer.

### 2.4. Data Extraction

Three independent reviewers (Xuan Ju, Zhenliang Jiang, and Jiayin Ma) completed relevant data extraction from each included report to reduce the reporting error, then extracted data were crossed over and any inconsistent differences were resolved through discussion until consensus. Extracted data included concentrations and proportions of fecal SCFAs as the main outcome parameters and basic characteristics of studies: (1) name of first author; (2) publication year; (3) country; (4) IBS subtypes; (5) diagnostic criteria of IBS; (6) participant information including age, sex, and number; (7) data format provided for SCFAs; (8) methods by which fecal SCFAs were analyzed.

### 2.5. Quality Assessment of Included Studies

Newcastle–Ottawa Scale (NOS) was used to evaluate the quality of included case–control studies, including three aspects: selection, comparability, and exposure [22]. The selection criteria contained adequate definition of cases, representativeness of cases, selection of controls, and definition of controls; the comparability criteria included comparability of cases and controls based on the design or analysis; the exposure criteria contained ascertainment of exposure, same method of ascertainment for cases and controls, and nonresponse rate. The sum of scores was equal to the total NOS score, where ≥7 means high quality.

RCTs were assessed using the Revised Cochrane Risk of Bias tool (RoB 2) [23]. Studies were classified as “low risk”, “high risk”, and “uncertain risk” based on seven bias criteria: random sequence generation, allocation concealment, blinding of participants and personnel, blinding of outcome assessment, incomplete outcome data, selective reporting, and other bias. The risk of bias summary was generated with Review Manger (RevMan) 5.4.1 software (Cochrane, Oxford, UK).

### 2.6. Statistical Analysis

RevMan was used for statistical analysis of continuous data available in included studies. MD values when outcomes were in the same unit or SMD values when not in the same unit with 95% CI, calculated from mean and standard deviation (SD), were used as a measure of effect. As for studies where results were presented as median, upper quartile, lower quartile, or interquartile range (IQR), mean and SD were estimated according to Wan’s method; as for those in which mean and 95% CI were extracted, SD was calculated using the method described in the Cochrane Handbook [24,25]. Heterogeneity across studies was examined by the *I*^2^ and *Q* test [26]. An *I*^2^ value < 50% and *p*-value > 0.1 were considered low-heterogeneity, with fixed effects model being used. Otherwise, the random effects model was used. Subgroup analysis of different IBS subtypes was performed to explore possible causes of statistical heterogeneity. A *p*-value < 0.05 was considered statistically significant in all analyses.

### 2.7. Sensitivity Analysis and Publication Bias Assessment

Stata/MP 17.0 software (StataCorp, College Station, TX, USA) was used for sensitivity analysis to show how the values of different studies affect the synthesis results. Funnel plots and Egger’s regression test were used to assess publication bias where potential publication bias might result in asymmetry of funnel plots.

## 3. Results

### 3.1. Study Selection

**Figure 1:**
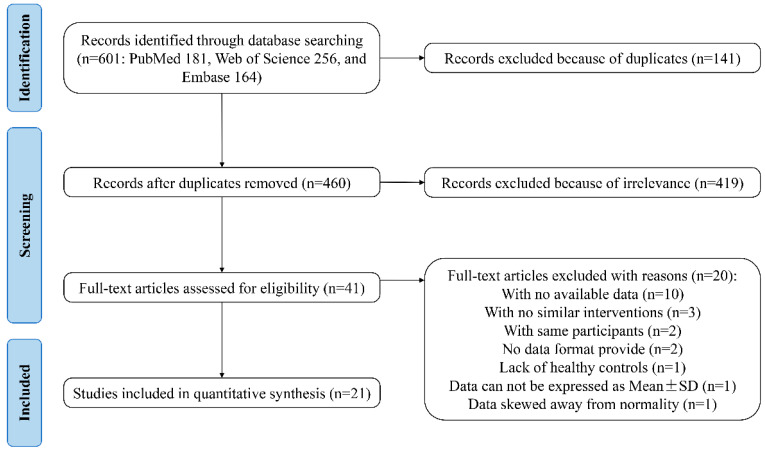
The PRISMA flowchart of assessment of studies identified in the meta-analysis. SD, standard deviation.

The workflow for the identification and stepwise selection of the studies is presented in Figure 1. Initially, a total of 601 citations (181 from PubMed, 256 from Web of Science, and 164 from Embase) were obtained through database searching. Among them, 141 records were removed for the reason of duplication and 460 articles were included for screening and filtering titles and abstracts, as described in Section 2. After excluding 419 irrelevant records, the remaining 41 articles were retrieved for a full-text review. Among them, 20 articles were excluded for various reasons: 10 failed to provide required data; 3 lacked similar interventions; 2 used the same participants; 2 did not indicate the data format; 1 lacked healthy controls; 1 provided data that cannot be expressed as mean ± SD; and 1 provided data skewed away from normality [16,27,28,29,30,31,32,33,34,35,36,37,38,39,40,41,42,43,44,45]. Finally, the cited references and citations of 21 remaining studies were manually searched, and no other eligible studies were included.

### 3.2. Study Characteristics

The characteristics of the included studies are summarized in Supplementary Tables S1 and S2. Of these 21 studies, 12 were case–control studies and 9 were self-controlled studies or RCTs [15,17,46,47,48,49,50,51,52,53,54,55,56]. Among the studies investigating the effects of interventions, there were three interventions: diets low in FODMAP, FMT, and supplementary probiotics. The publication year of the included studies varied from 1984 to 2023, and the geographical distribution covered four continents (13 conducted in Europe, 5 in Asia, 2 in North America, and 1 in Oceania). The available data of the fecal SCFAs in 10 studies were expressed in the format of mean ± SD, and in other studies were expressed as median (IQR), mean (95% CI), mean ± SEM, and estimated marginal mean (EMM) ± SEM. All the data in the other formats were converted into mean ± SD for synthesis in the meta-analysis. Concerning the methods for detecting fecal SCFAs, 10 studies used gas chromatography (GC), and other methods included gas–liquid chromatography (GLC), gas chromatography-mass spectrometry (GC-MS), and high-performance liquid chromatography (HPLC), etc.

### 3.3. Quality Assessment of Studies

As shown in Supplementary Table S3, we used NOS to evaluate the quality of each case–control study. Among them, 10 studies were sufficient to conduct a meta-analysis with moderate quality (NOS score ≥ 7), whereas the other 2 studies published in the 1980s were excluded because of their low quality. As shown in Supplementary Figure S1, the risk of bias in four RCTs was assessed, and the quality of the included RCTs was moderate. After removing 2 studies, the 10 case–control studies included 553 IBS patients and 258 HCs, and 225 IBS patients in treatment groups and 220 as controls were included.

### 3.4. Results of Synthesis

Our study focused on the changes in the fecal SCFA concentrations (including total SCFAs, acetate, propionate, butyrate, iso-butyrate, valerate, and iso-valerate) and each of their proportions in IBS patients and the effects of interventions. The results of our meta-analysis are displayed in forest plots.

**Figure 2:**
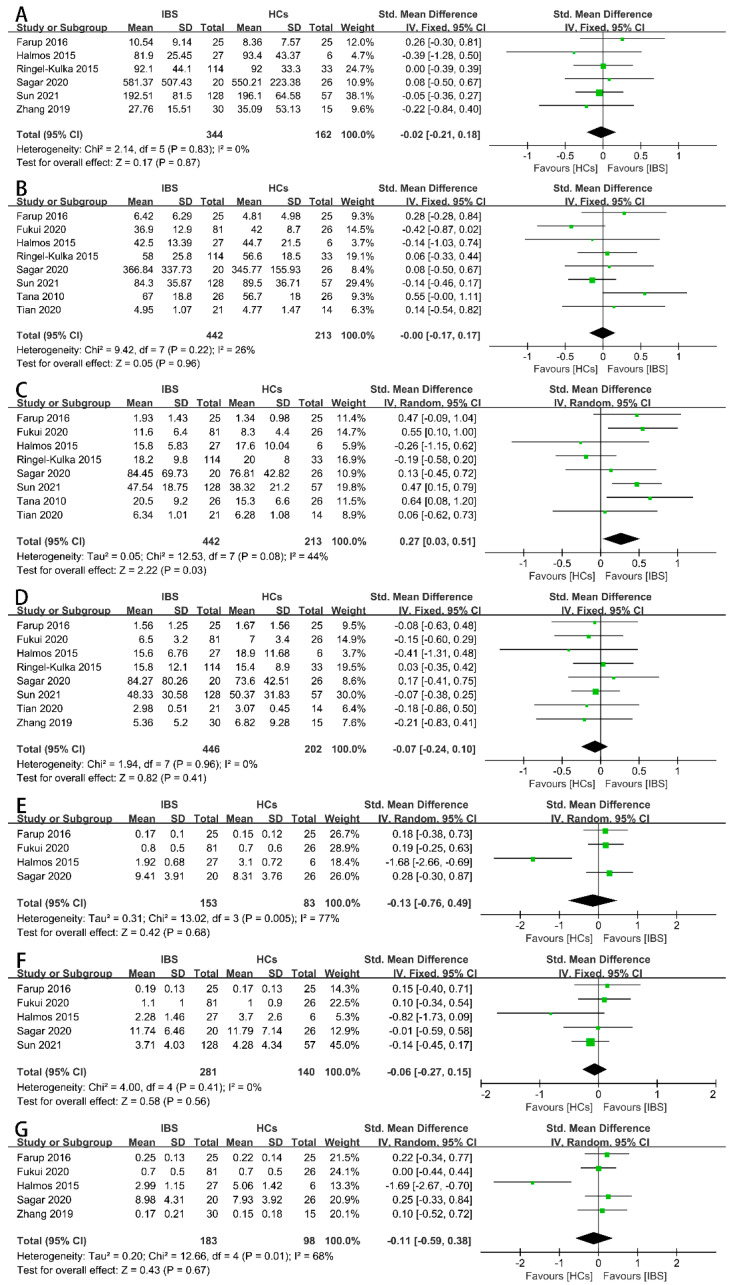
Forest plots of alterations of concentration of fecal SCFAs in IBS patients versus HCs: (**A**) total SCFAs, (**B**) acetate, (**C**) propionate, (**D**) butyrate, (**E**) iso-butyrate, (**F**) valerate, and (**G**) iso-valerate.

**Figure 3:**
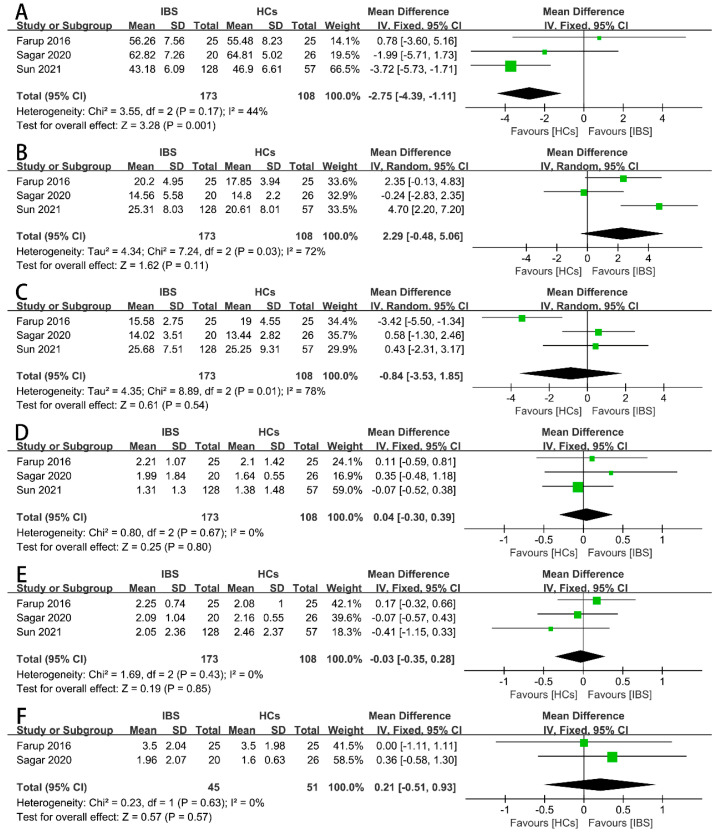
Forest plots of alterations of proportion of fecal SCFAs in IBS patients versus HCs: (**A**) acetate, (**B**) propionate, (**C**) butyrate, (**D**) iso-butyrate, (**E**) valerate, and (**F**) iso-valerate.

Firstly, we examined the concentrations of the total SCFAs (Figure 2A), acetate (Figure 2B), propionate (Figure 2C), butyrate (Figure 2D), iso-butyrate (Figure 2E), valerate (Figure 2F), and iso-valerate (Figure 2G) in the feces of IBS patients and HCs as possible indicators. Nine case–control studies with 472 IBS patients and 228 HCs showed that the concentration of fecal propionate in the IBS patients was significantly higher than that in the HCs (SMD = 0.27, 95% CI 0.03 to 0.51, *p* < 0.05), and the heterogeneity among the included studies was significantly different (*p* = 0.08, *I*^2^ = 44%). None of the other indicators exhibited a significant correlation with IBS patients or HCs. The proportions of SCFAs in the total concentration of SCFAs in the IBS patients and HCs were further examined. Three studies including 173 IBS patients and 108 HCs showed that the proportions of fecal propionate (Figure 3B), butyrate (Figure 3C), iso-butyrate (Figure 3D), valerate (Figure 3E), and iso-valerate (Figure 3F) were not significantly different among the IBS patients or HCs. Except the proportion of fecal acetate was significantly lower compared with the HCs (Figure 3A, MD = −2.75, 95% CI −4.39 to −1.11, *p* < 0.05), the heterogeneity among the included studies was not significantly different (*p* = 0.17, *I*^2^ = 44%).

**Figure 4:**
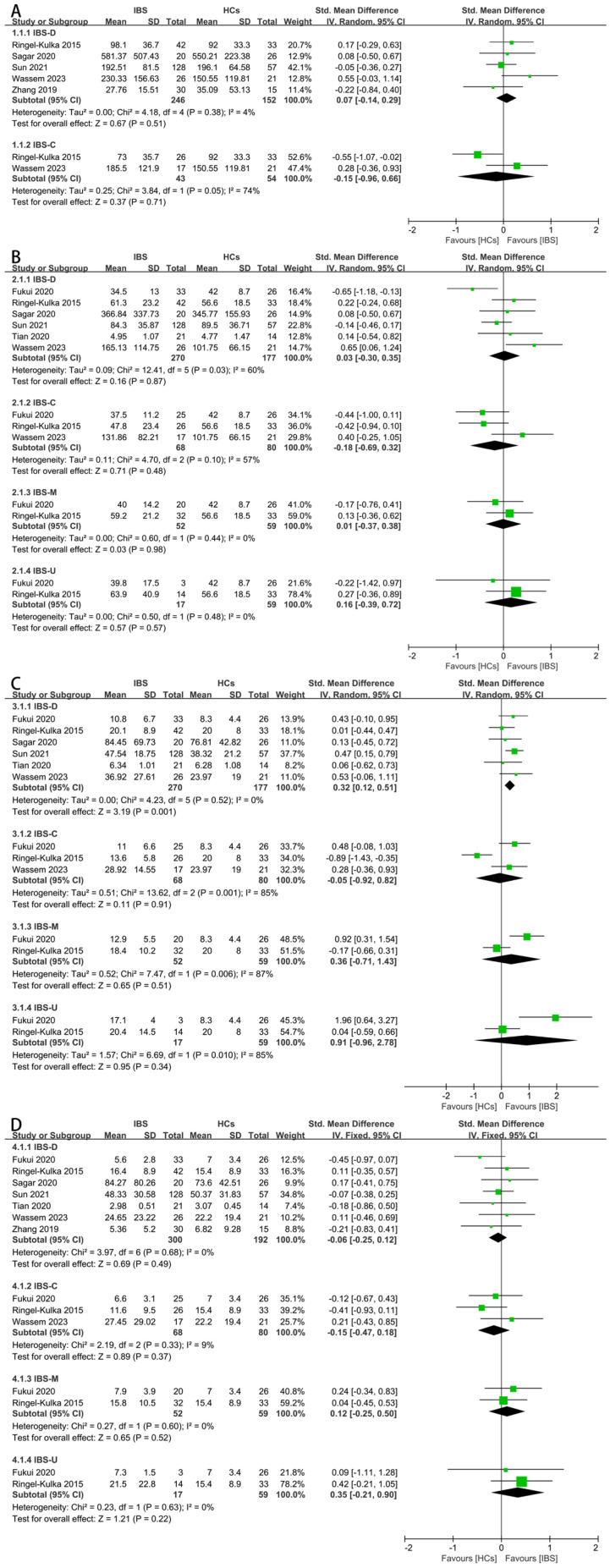
Forest plots of alterations of concentration of fecal SCFAs in IBS subtypes versus HCs: (**A**) total SCFAs, (**B**) acetate, (**C**) propionate, and (**D**) butyrate.

Next, a subgroup analysis based on the different IBS subtypes were conducted. As for the total SCFAs, there was no significant difference between the IBS-D patients or IBS-C patients and HCs (Figure 4A). As for acetate, there was no significant difference between the four subtypes (Figure 4B). As for propionate, the fecal concentration between the IBS-D patients and HCs was significantly different (Figure 4C, SMD = 0.32, 95% CI 0.12 to 0.51), and the result of this subgroup analysis was consistent with the overall results of the IBS patients. For butyrate, the fecal concentrations were found to be irrelevant among the different subtypes of IBS and HCs (Figure 4D). The concentrations of iso-butyrate (Supplementary Figure S2A), valerate (Supplementary Figure S2B), and iso-valerate (Supplementary Figure S2C) in the feces of the IBS-D patients and HCs were examined since the other subtypes lacked corresponding data, and there were no significant differences.

The effects of three interventions: low-FODMAP diets, FMT, and supplementary probiotics were studied. As for the self-controlled studies, the concentrations of fecal SCFAs in the IBS patients before and after receiving the interventions were the major outcome measure, and the baseline-adjusted marginal means of the concentration of fecal SCFAs in the treatment group and placebo group were the major outcome measure in the RCTs. Diets low in FODMAP significantly reduced the concentrations of fecal total SCFAs (Supplementary Figure S3A, SMD = −0.41, 95% CI −0.69 to −0.13, *p* < 0.05), acetate (Supplementary Figure S3B, SMD = −0.41, 95% CI −0.66 to −0.15, *p* < 0.05), propionate (Supplementary Figure S3C, SMD = −0.29, 95% CI −0.55 to −0.04, *p* < 0.05), and butyrate (Supplementary Figure S3D, SMD = −0.32, 95% CI −0.58 to −0.07, *p* < 0.05). There were no significant differences in the other indicators. FMT did not significantly change the concentrations of fecal total SCFAs, acetate, propionate, and butyrate (Supplementary Figure S4A–D), but the concentrations of iso-butyrate (Supplementary Figure S4E, SMD = 0.30, 95% CI 0.02 to 0.59, *p* < 0.05), valerate (Supplementary Figure S4F, SMD = 0.24, 95% CI 0.07 to 0.41, *p* < 0.05), and iso-valerate (Supplementary Figure S4G, SMD = 0.48, 95% CI 0.08 to 0.89, *p* < 0.05) were significantly different from the controls. Supplementary probiotics did not exhibit a significant impact on the fecal concentrations of any SCFAs in the IBS patients (Supplementary Figure S5).

### 3.5. Publication Bias

**Figure 5:**
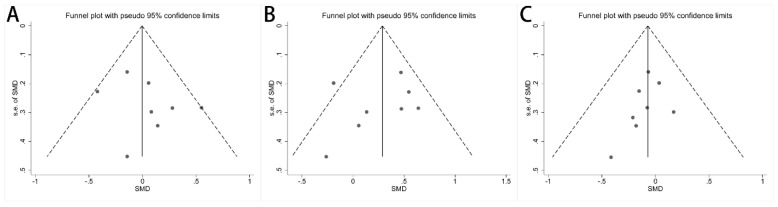
Funnel plots of alterations of concentration of fecal SCFAs in IBS patients versus HCs: (**A**) acetate, (**B**) propionate, and (**C**) butyrate.

Publication bias among the included studies was assessed and is shown in funnel plots. There was no publication bias for the concentration of acetate (Figure 5A, *p* = 0.354), propionate (Figure 5B, *p* = 0.585), and butyrate (Figure 5C, *p* = 0.287). The publication bias for the other fecal SCFA concentrations was assessed by Egger’s test, and no publication bias was found (Supplementary Table S4). A sensitivity analysis was performed by culling individual studies to analyze the effect on the synthesis result. The synthesis results did not change significantly after removing each study (Supplementary Figure S6 and Supplementary Table S5).

## 4. Discussion

Gut microbiota dysbiosis contributes to the development of intestinal disorders, which is confirmed by clinical and experimental evidence [6]. Gut microbiota dysbiosis induces pathophysiological reactions such as activation of the mucosal immune system, increased intestinal permeability, activation of sensory pathways, and modulation of the enteric motility [46]. Several studies show evidence for the association and differences between the gut microbiota in IBS patients and HCs [57,58,59]. SCFAs are produced by gut microbiota through the fermentation of ingestible polysaccharides and proteins, and have been described as the link between the microbes and the host [60]. SCFAs exhibit anti-inflammatory effects through the modulation of immune cell chemotaxis, and the release of reactive oxygen species (ROS) and cytokines [61]. The effects are mediated mainly by the inhibition of histone deacetylases (HDACs) and stimulation of G-protein-coupled receptors (GPCRs), particularly GPR43 [62]. Studies have shown the activation of the immune system and an imbalance in the cytokine pattern in IBS patients, indicating IBS might be an inflammation-mediated disease [63]. Acetate has the highest concentration than the other SCFAs, and is involved in the carbohydrate and fat metabolic pathways [64]. Propionate is a potent activator of GPCR43 that is present in immune, nervous, and endocrine cells along the entire gastrointestinal tract [65]. Butyrate, which is a key promoter of colonic health and the main provider of energy for colonocytes, inhibits Il-12 and increases Il-10 production [66]. Considering the significance of the correlation between SCFAs and IBS, it is urgently needed to explore consistent SCFA characterization in IBS patients and identify whether fecal SCFAs could be a valuable biomarker for IBS diagnosis.

In our analysis, the fecal propionate concentration in IBS patients was found to be significantly higher than that in HCs, suggesting that it can serve as a biomarker for IBS diagnosis. The increase in propionate is consistent with a previous report, which revealed that the propionate-producing genera *Lactobacillus* and *Veillonella* are increased in IBS patients, and they transform lactic acid into propionate [15,67]. And it is speculated that sustained acid stimuli in the gut mucosa leads to the activation of subcortical brain nuclei that are involved in emotional, behavioral, neuroendocrine, autonomic, and antinociceptive reactions to noxious stimuli [68]. Another explanation is that higher amounts of SCFAs in the feces are associated with less absorption, which results in gut dysbiosis, gut permeability, excess adiposity, and cardiometabolic risk factors [69]. The concentrations of fecal acetate in two studies were lower than those in HCs, while another study showed the opposite result [15,47,52]. And there are no significant differences in the synthesis results of the concentrations of the other fecal SCFA indicators between the IBS patients and HCs. Most studies used the symptom-based Rome criteria for the diagnosis of IBS, and the results indicate that the concentration of fecal propionate could be a reliable biomarker for the differentiation of healthy subjects from subjects with IBS. The relative amount of each SCFA is also important, and the ratio of acetate, propionate, and butyrate in HCs is approximately 5:2:2 [70]. Three of the included studies reported the proportion of fecal SCFAs in the IBS patients, and the result shows that the proportion of fecal acetate in the IBS patients is significantly lower than that in the HCs [46,51,52]. Our conclusion is in contrast with previous studies, mainly because the limited publications included in their data synthesis (*n* = 5 and *n* = 15), and more than half of the studies included in our study were published after these previous studies [71,72].

IBS is conventionally classified into four subtypes according to the associated bowel habits: IBS-D, IBS-C, IBS-M, and IBS-U. The diverse mechanisms underlying the pathophysiology of the IBS subtypes remain unknown, and validated mechanistic biomarkers for the subtypes are not available [28]. A detailed examination is performed to further distinguish the relation between the IBS subtypes and SCFA concentrations, while the concentration data of the total SCFAs, iso-butyrate, valerate, and iso-valerate is only available for the IBS-D subtype. For the IBS-C, IBS-M, and IBS-U subtypes, data on the acetate, propionate, and butyrate concentrations are available. In the subgroup analysis, the concentration of fecal propionate in the IBS-D patients is significantly higher than that in HCs, consistent with the result of the entire IBS patient analysis. IBS-C patients exhibit a significantly lower amount of fecal acetate than HCs. Compared to IBS-C, IBS-D patients exhibit increased intestinal motility and decreased transit time, so the results can be explained by two possible mechanisms: (1) colonic fermentation is stimulated by increased intestinal motility, leading to higher fecal levels of SCFAs; and (2) the decreased transit time slows down the absorption of SCFAs, contributing to the accumulation of SCFAs [73]. Fecal SCFAs including the proportion of acetate and the concentration of propionate can be used as biomarkers for distinguishing the IBS subtypes.

Among these treatments analyzed, diets low in FODMAP lead to a significant reduction in the abnormal fecal total SCFA, acetate, and propionate concentrations in the IBS patients compared with the pre-treatment patients or those receiving a placebo, indicating the low-FODMAP diet normalized the IBS patients’ abnormal SCFA concentrations. Further studies need to assess if this normalization is linked to a clinical improvement and its efficacy as an intervention for IBS patients, which is quite promising, as suggested by previous findings [36,74]. While other studies found that the low-FODMAP diet did not significantly alter the fecal SCFAs [75,76], it is possible that low FODMAP administration to IBS patients should be offered with personalization and the later reintroduction of FODMAPs after the alleviation of IBS symptoms [36,77].

On the other hand, FMT increases the concentrations of iso-butyrate, valerate, and iso-valerate in IBS patients. While there is no significant difference in fecal iso-butyrate, valerate, and iso-valerate between IBS patients and HCs, FMT seems unhelpful for IBS patients. This is consistent with the results of a previous study, where no significant difference in the global improvement in IBS symptoms was observed at 12 weeks with FMT vs. placebo [78]. Other studies showed very-low-quality evidence to support recommending FMT for IBS, which requires larger and more rigorously conducted trials [79,80]. Currently, FMT is only recommended for the treatment of *Clostridioides difficile* infection and should be applied with extreme caution for other conditions [81]. There are also no significant changes in the SCFA concentrations after supplementary probiotics, and this is highly likely due to the absence of probiotic strain information in the studies included. It is reported that single-strain probiotics or their mixtures showed significant efficacy for at least one IBS outcome measure [82]. A more robust clinical trial for probiotic administration for treating IBS is needed.

## 5. Conclusions

In conclusion, there is a correlation between IBS and the concentration of fecal SCFAs including propionate and acetate, so these two could be used as biomarkers for IBS diagnosis. Specifically, the fecal propionate concentration could be used as a biomarker for IBS-D subtype diagnosis. A diet low in FODMAPs is an effective intervention for IBS patients, while FMT or supplementary probiotics seem unhelpful for IBS patients.

## Data Availability

No new data were created.

## Supplementary Materials

The following supporting information can be downloaded at https://www.mdpi.com/article/10.3390/nu16111727/s1. Supplementary method, search strategy. Figure S1: Risk of bias for treatment studies. Figure S2: Forest plots of alterations of concentration of fecal SCFAs in IBS-D patients versus HCs. Figure S3: Forest plots of alterations of concentration of fecal SCFAs in IBS patients after treatment with diets low in FODMAP. Figure S4: Forest plots of alterations of concentration of fecal SCFAs in IBS patients after FMT treatment. Figure S5: Forest plots of alterations of concentration of fecal SCFAs in IBS patients after supplementary probiotic treatment. Figure S6: Results of sensitivity analysis. Table S1: Characteristics of included case–control studies. Table S2: Characteristics of included self-controlled studies and randomized controlled trials. Table S3: Quality assessment of included case–control studies (*n* = 12) using Newcastle–Ottawa Scale (NOS). Table S4: Results of Egger’s test. Table S5: Results of sensitivity analysis.
