# Supplementary material for: Changes in Fecal Short-Chain Fatty Acids in IBS Patients and Effects of Different Interventions: A Systematic Review and Meta-Analysis
Source: Nutrients. 2024 May 31;16(11):1727. doi: 10.3390/nu16111727 (PMC11174707; doi:10.3390/nu16111727)
Supplement: Supplementary file 1 [file nutrients-16-01727-s001.zip › nutrients-3021610-supplementary.pdf]

Supplementary Figures

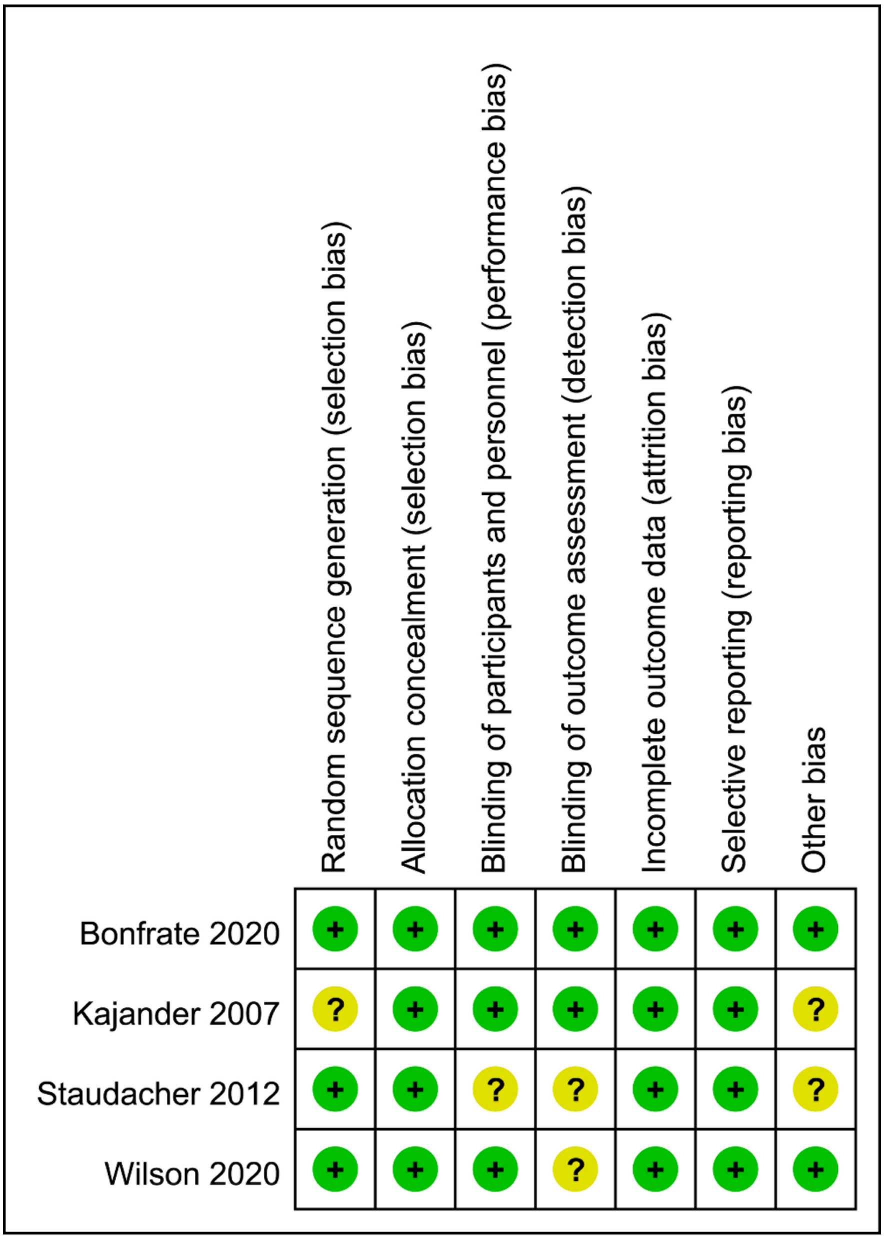

Figure S1 Risk of bias for treatment studies.

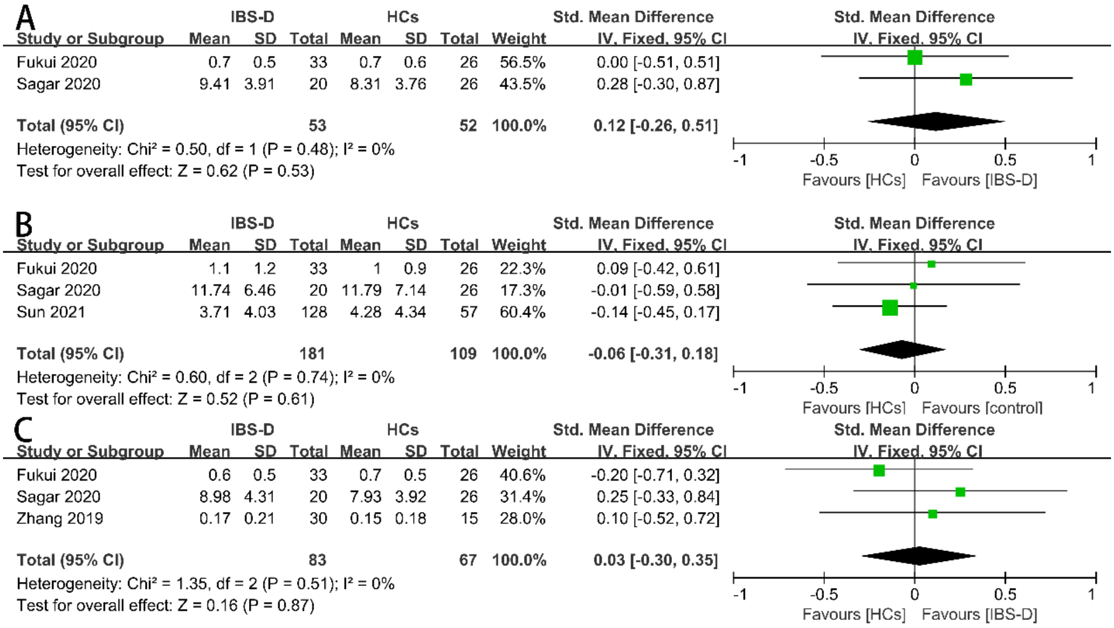

Figure S2 Forest plots of alterations of concentration of fecal SCFAs in IBS-D patients versus HCs: (A) iso-butyrate, (B) valerate, and (C) iso-valerate.

A

Study or Subgroup	Experimental Mean	Experimental SD	Experimental Total	Control Mean	Control SD	Control Total	Weight	Std. Mean Difference IV, Fixed, 95% CI
Staudacher 2012	140	56.12	16	136	55.6	19	17.6%	0.07 [-0.60, 0.74]
Valeur 2016	73.7	27.3	63	87.1	32.6	63	62.4%	-0.44 [-0.80, -0.09]
Wilson 2020	2,189.9	1,274.87	21	3,150.1	1,352.78	21	19.9%	-0.72 [-1.34, -0.09]
**Total (95% CI)**			**100**			**103**	**100.0%**	**-0.41 [-0.69, -0.13]**

Heterogeneity: Chi² = 2.96, df = 2 (P = 0.23); I² = 32%
Test for overall effect: Z = 2.86 (P = 0.004)

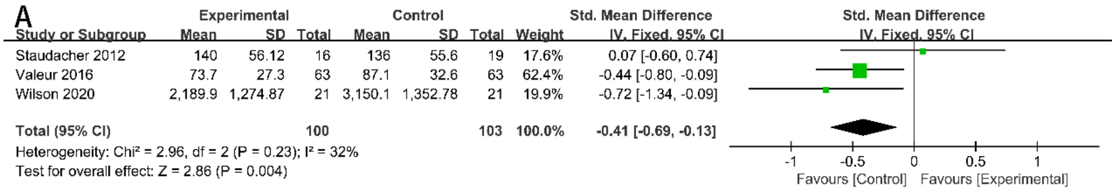

B

Study or Subgroup	Experimental Mean	Experimental SD	Experimental Total	Control Mean	Control SD	Control Total	Weight	Std. Mean Difference IV, Fixed, 95% CI
Caponio 2022	6.88	11.53	18	9.46	9.04	18	15.4%	-0.24 [-0.90, 0.41]
Staudacher 2012	81	31.63	16	81	31.13	19	14.9%	0.00 [-0.67, 0.67]
Valeur 2016	43.2	16.5	63	52.7	20.9	63	52.5%	-0.50 [-0.86, -0.15]
Wilson 2020	1,263.8	757.5	21	1,753	803.78	21	17.2%	-0.61 [-1.23, 0.01]
**Total (95% CI)**			**118**			**121**	**100.0%**	**-0.41 [-0.66, -0.15]**

Heterogeneity: Chi² = 2.38, df = 3 (P = 0.50); I² = 0%
Test for overall effect: Z = 3.10 (P = 0.002)

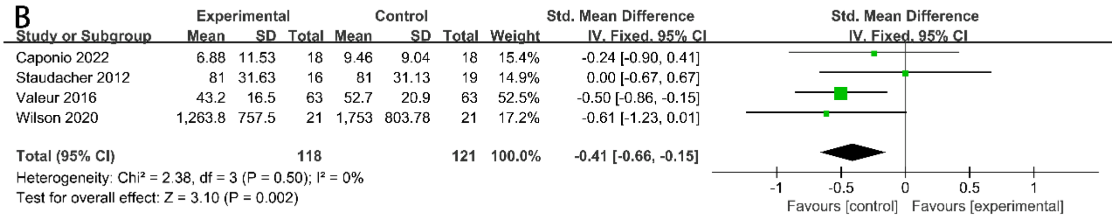

C

Study or Subgroup	Experimental Mean	Experimental SD	Experimental Total	Control Mean	Control SD	Control Total	Weight	Std. Mean Difference IV, Fixed, 95% CI
Caponio 2022	4.16	4.95	18	5.86	6.91	18	15.2%	-0.28 [-0.93, 0.38]
Staudacher 2012	25	13.27	16	24	14.46	19	14.8%	0.07 [-0.60, 0.74]
Valeur 2016	12.5	5.9	63	14	6.2	63	53.4%	-0.25 [-0.60, 0.10]
Wilson 2020	379.8	292.83	21	624.2	309.78	21	16.5%	-0.80 [-1.43, -0.16]
**Total (95% CI)**			**118**			**121**	**100.0%**	**-0.29 [-0.55, -0.04]**

Heterogeneity: Chi² = 3.65, df = 3 (P = 0.30); I² = 18%
Test for overall effect: Z = 2.25 (P = 0.02)

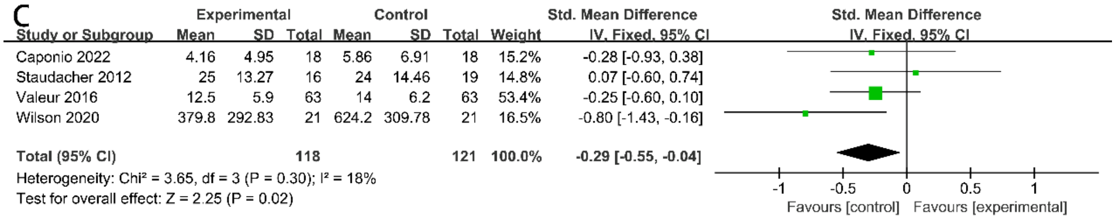

D

Study or Subgroup	Experimental Mean	Experimental SD	Experimental Total	Control Mean	Control SD	Control Total	Weight	Std. Mean Difference IV, Fixed, 95% CI
Caponio 2022	14.8	27.13	18	17.9	14.57	18	15.4%	-0.14 [-0.79, 0.52]
Staudacher 2012	26	13.27	16	23	13.34	19	14.8%	0.22 [-0.45, 0.89]
Valeur 2016	11.6	6	63	14.4	8.6	63	53.2%	-0.38 [-0.73, -0.02]
Wilson 2020	387.3	257.54	21	609.2	274.04	21	16.5%	-0.82 [-1.45, -0.19]
**Total (95% CI)**			**118**			**121**	**100.0%**	**-0.32 [-0.58, -0.07]**

Heterogeneity: Chi² = 5.30, df = 3 (P = 0.15); I² = 43%
Test for overall effect: Z = 2.47 (P = 0.01)

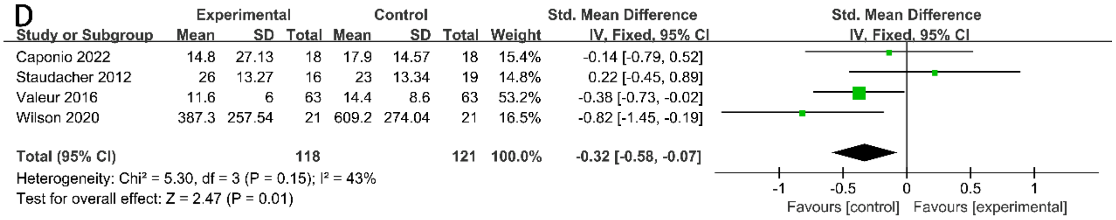

E

Study or Subgroup	Experimental Mean	Experimental SD	Experimental Total	Control Mean	Control SD	Control Total	Weight	Std. Mean Difference IV, Fixed, 95% CI
Caponio 2022	1.73	1.47	18	2.41	2.54	18	14.9%	-0.32 [-0.98, 0.34]
Staudacher 2012	2	1.02	16	2	2.22	19	14.6%	0.00 [-0.67, 0.67]
Valeur 2016	1.7	0.8	63	1.6	0.8	63	52.8%	0.12 [-0.23, 0.47]
Wilson 2020	50.8	24.29	21	48.9	25.2	21	17.6%	0.08 [-0.53, 0.68]
**Total (95% CI)**			**118**			**121**	**100.0%**	**0.03 [-0.22, 0.29]**

Heterogeneity: Chi² = 1.40, df = 3 (P = 0.71); I² = 0%
Test for overall effect: Z = 0.24 (P = 0.81)

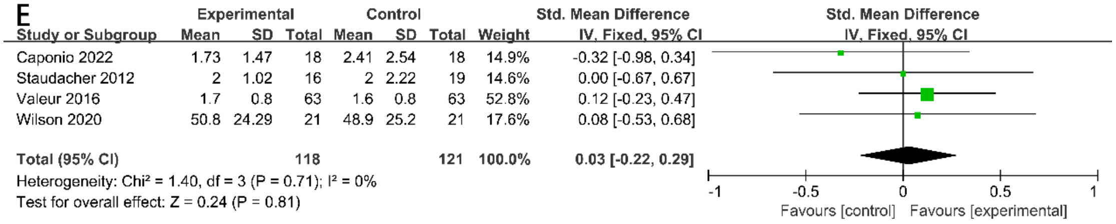

F

Study or Subgroup	Experimental Mean	Experimental SD	Experimental Total	Control Mean	Control SD	Control Total	Weight	Std. Mean Difference IV, Fixed, 95% CI
Caponio 2022	8.53	9.38	18	10.1	8.81	18	15.2%	-0.17 [-0.82, 0.49]
Staudacher 2012	2	1.02	16	2	1.11	19	14.7%	0.00 [-0.67, 0.67]
Valeur 2016	1.7	0.8	63	1.7	0.7	63	53.4%	0.00 [-0.35, 0.35]
Wilson 2020	49.6	29.33	21	70.8	30.7	21	16.7%	-0.69 [-1.32, -0.07]
**Total (95% CI)**			**118**			**121**	**100.0%**	**-0.14 [-0.40, 0.11]**

Heterogeneity: Chi² = 3.80, df = 3 (P = 0.28); I² = 21%
Test for overall effect: Z = 1.09 (P = 0.28)

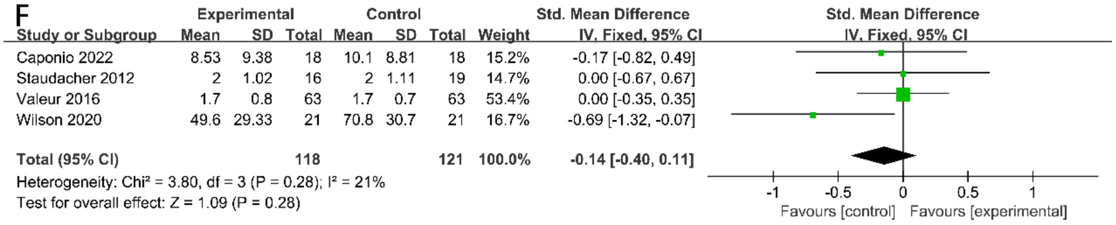

G

Study or Subgroup	Experimental Mean	Experimental SD	Experimental Total	Control Mean	Control SD	Control Total	Weight	Std. Mean Difference IV, Fixed, 95% CI
Caponio 2022	8.93	8.11	18	13.1	12.83	18	14.9%	-0.38 [-1.04, 0.28]
Staudacher 2012	3	2.04	16	3	2.22	19	14.7%	0.00 [-0.67, 0.67]
Valeur 2016	2.4	1.1	63	2.1	1.8	63	52.9%	0.20 [-0.15, 0.55]
Wilson 2020	62.5	26.12	21	56.9	27.5	21	17.6%	0.20 [-0.40, 0.81]
**Total (95% CI)**			**118**			**121**	**100.0%**	**0.09 [-0.17, 0.34]**

Heterogeneity: Chi² = 2.53, df = 3 (P = 0.47); I² = 0%
Test for overall effect: Z = 0.66 (P = 0.51)

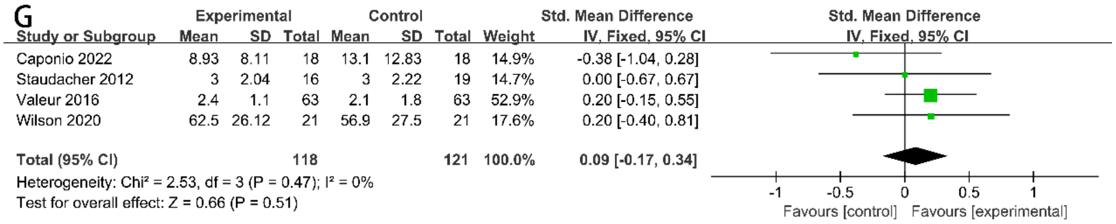

Figure S3 Forest plots of alterations of concentration of fecal SCFAs in IBS patients after diets low in FODMAP treatment: (A) total SCFAs, (B) acetate, (C) propionate, (D) butyrate, (E) iso-butyrate, (F) valerate, and (G) iso-valerate.

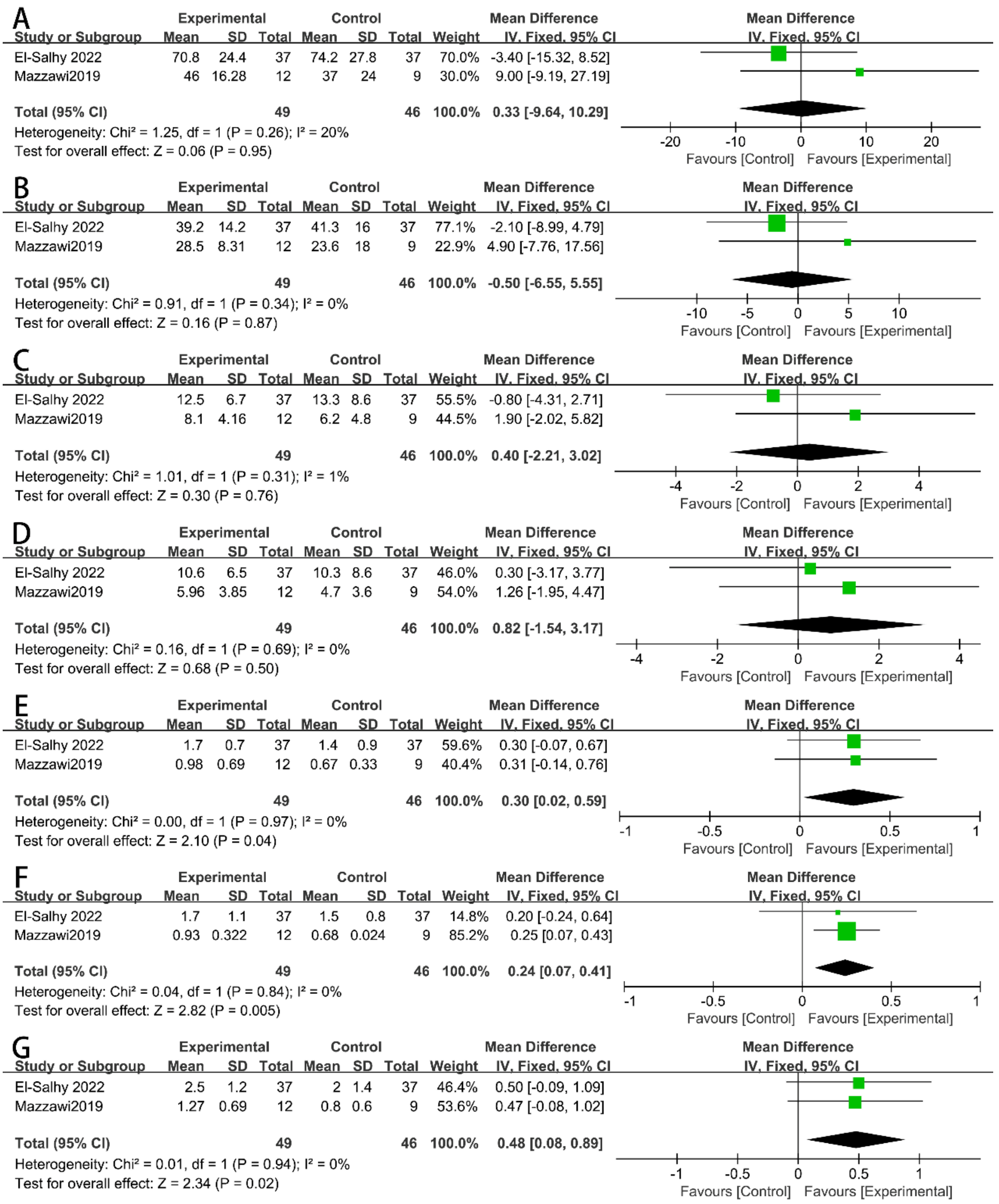

Figure S4 Forest plots of alterations of concentration of fecal SCFAs in IBS patients after FMT treatment: (A) total SCFAs, (B) acetate, (C) propionate, (D) butyrate, (E) iso-butyrate, (F) valerate, and (G) iso-valerate.

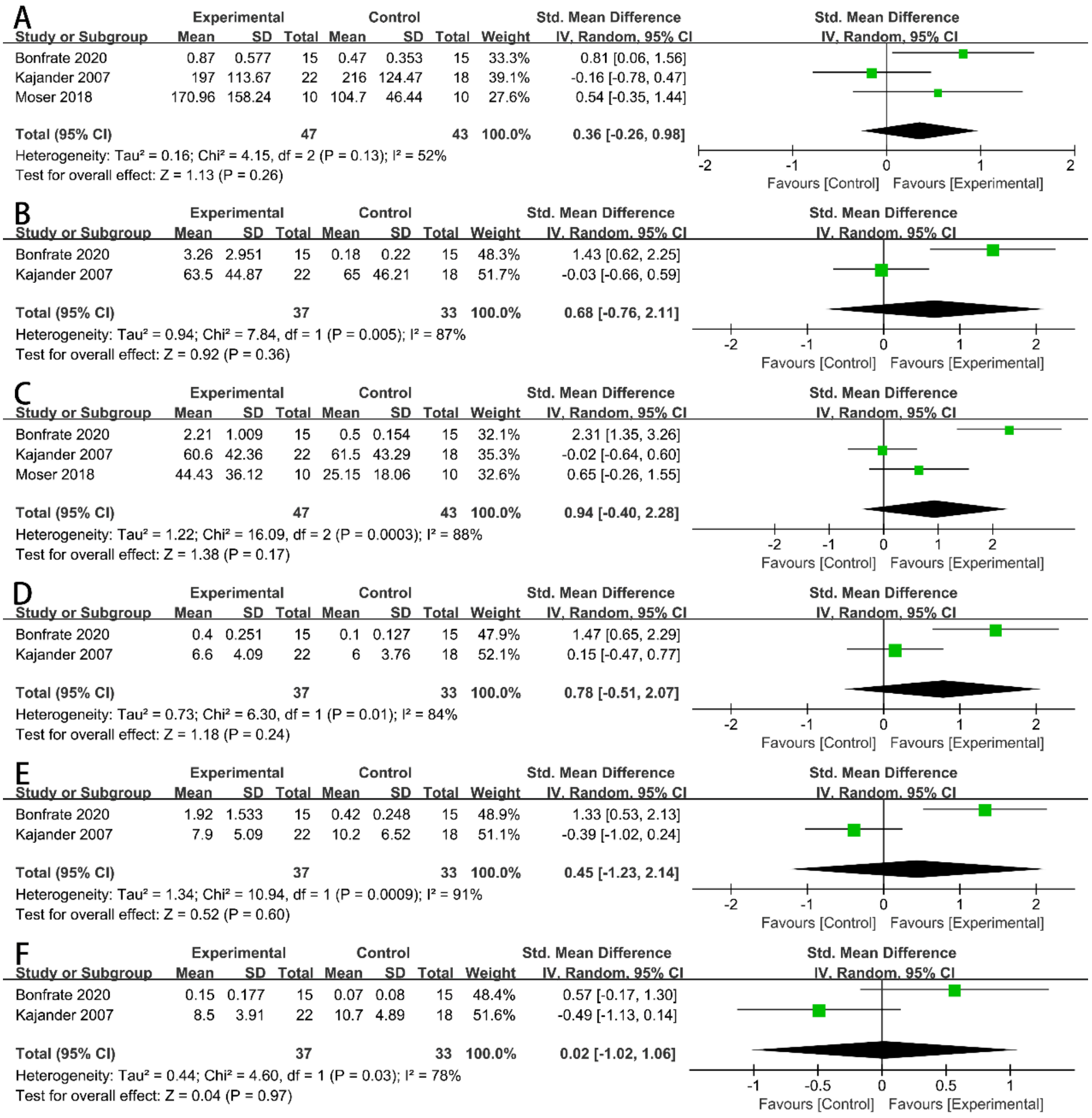

Figure S5 Forest plots of alterations of concentration of fecal SCFAs in IBS patients after probiotics supplementary treatment: (A) total SCFAs, (B) acetate, (C) propionate, (D) butyrate, (E) iso-butyrate, and (F) valerate.

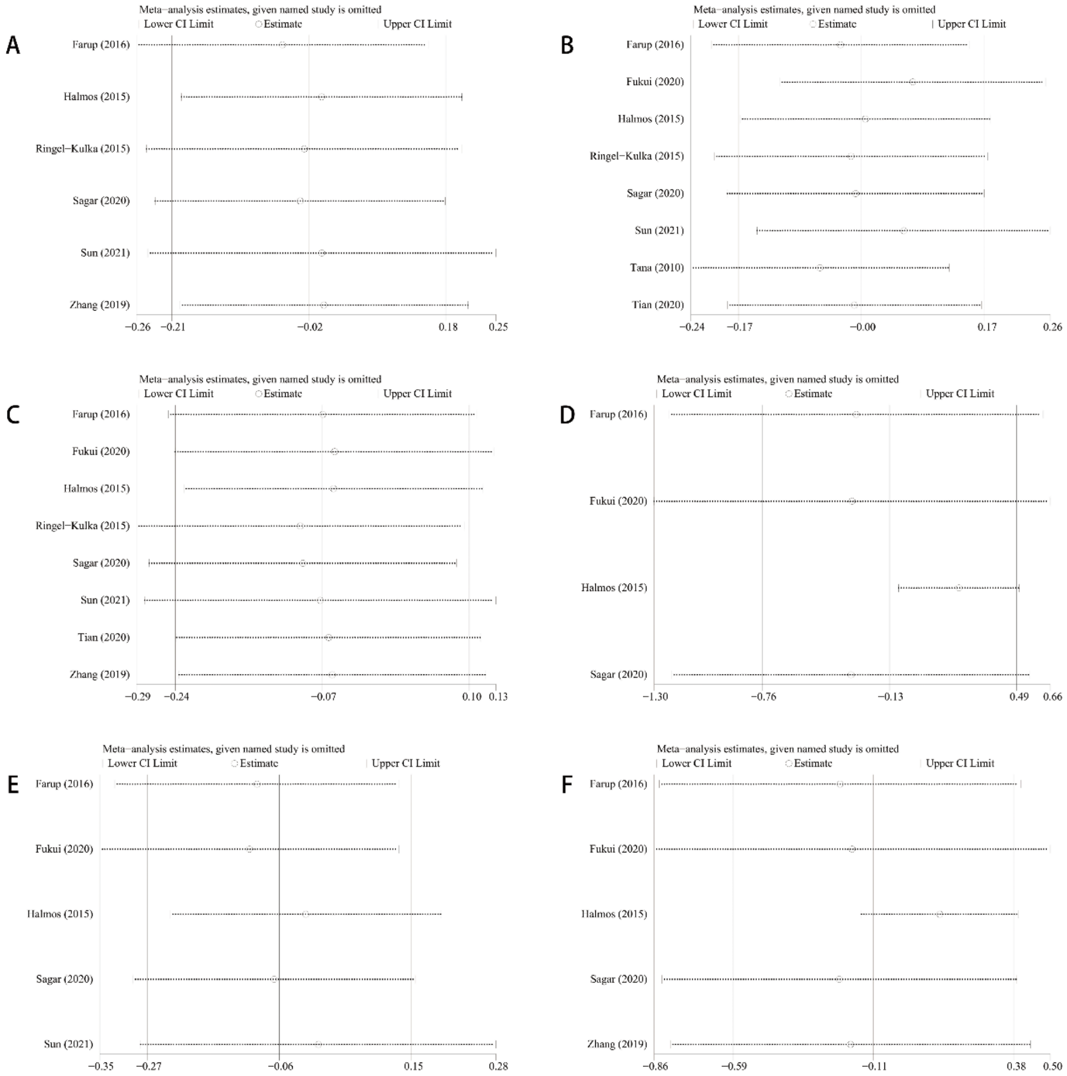

Figure S6 Results of sensitivity analysis: (A)total SCFAs, (B) acetate, (C) butyrate, (D) iso-butyrate, (E) valerate, (F) iso-valerate.

Supplementary Table S1 Characteristics of included case-control studies.

Reference	Year	Country	Diagnostic criteria	IBS/HC	Age (Mean, SD)		Sex (M/F)		Data format	Method
					IBS	HC	IBS	HC		
Farup	2016	Norway	Rome III	25/25	46.2, 12.9	49.2, 14.6	12/13	10/15	Mean, SD	GC
Fukui	2020	Japan	Rome III	85/26	51.3, 15.3	46.2, 10.6	37/48	9/17	Mean, SD	GC-MS
Halmos	2015	Australia	Rome III	27/6	41.9, 19.6	39.4, 36.2	6/21	1/5	Mean, 95% CI	GC
Mortensen	1987	Denmark	No data	18/9	44.9, 11.5	No data	4/14	No data	Mean, SD	GLC
Ringel-Kulka	2015	USA	Rome III	114/33	35.4, 11.3	33.9, 13.0	16/98	4/29	Mean, SD	GC
Sagar	2020	UK	Rome III	20/26	55.4, 13.1	55.4, 13.1	3/17	11/15	Median, IQR	GC
Sun	2021	China	Rome III	162/66	35.5, 11.7	36.7, 11.8	114/48	38/28	Median, IQR	UPLC-MS/MS
Tana	2010	Japan	Rome II	26/26	21.7, 2.0	21.9, 2.9	13/13	13/13	Mean, SD	HPLC
Tian	2020	China	Rome IV	21/14	31.6, 8.9	27.0, 4.4	13/8	8/6	Mean, SD	HS-SPME-GC-MS
Vernia	1987	Italy	No data	38/50	38.2, 14.0	38.2, 12.0	No data	No data	Mean, SD	GLC
Waseem	2023	USA	Rome IV	43/21	No data	32.1, 12.8	10/33	5/16	Median, IQR	HPLC
Zhang	2019	China	Rome IV	30/15	30 (28, 41)[‡]	28 (25, 37)[‡]	22/8	11/4	Median, IQR	GC-MS

Abbreviations: GC, gas chromatography; GC-MS, gas chromatography-mass spectrometry; GLC, gas-liquid chromatography; UPLC-MS/MS, ultra performance liquid chromatography-tandem mass spectrometry; HPLC, high performance liquid chromatography; HS-SPME-GC-MS, headspace solid-phase microextraction coupled with gas chromatography−mass spectrometry.

[†]Mean (range) [‡]Median (IQR)

Supplementary Table S2 Characteristics of included self-controlled studies and randomized controlled trials

Reference	Year	Country	Treatment	Study design	Diagnostic criteria	Treatment/Control	Age (Mean, SD)		Sex (M/F)		Data format	Method
							Treatment	Control	Treatment	Control		
Caponio	2022	Italy	Low FODMAP diet	Self-controlled study	Rome Ⅳ	18/18	42.9, 10.2	42.9, 10.2	4/14	4/14	Mean, SEM	GC
Staudacher	2012	UK	Low FODMAP diet	RCT	Rome Ⅲ	16/19	36.4, 11.6	35.5, 9.1	5/11	7/12	Mean, 95% CI	GLC
Valeur	2016	Norway	Low FODMAP diet	Self-controlled study	Rome Ⅲ	63/63	38 (19-67)†	38 (19-67)†	7/56	7/56	Mean, SD	GC
Wilson	2020	UK	Low FODMAP diet	RCT	Rome Ⅲ	22/23	38.9, 10.0	30.3, 9.8	9/13	11/12	EMM, SEM	GLC
El-Salhy	2022	Norway	FMT	Self-controlled study	Rome Ⅳ	40/40	38.1, 14.4	38.1, 14.4	33/7	33/7	Mean, SD	GC
Mazzawi	2019	Norway	FMT	Self-controlled study	Rome Ⅲ	13/13	32 (20-44)†	32 (20-44)†	4/9	4/9	Mean, SEM	GC

Bonfrate	2020	Italy	Probiotics	RCT	Rome Ⅳ	15/10	50, 11	46, 10	3/12	4/6	Mean, SD	GC
Kajander	2007	Finland	Probiotics	RCT	Rome Ⅰ, Ⅱ	28/27	44 (23-65)†	47 (21-65)†	9/19	7/20	Mean, 95% CI	GC
Moser	2018	Austria	Probiotics	Self-controlled study	No data	10/10	45.3, 13.8	45.3, 13.8	5/5	5/5	Median, IQR	GC-MS

Abbreviations: EMM, estimated marginal mean

†Mean (range)

Supplementary Table S3 Quality assessment of included case-control studies (n=12) using Newcastle-Ottawa Scale (NOS)

Study	Selection (4)				Comparability (2)		Exposure (3)			Total scores
	**Adequate definition of cases**	**Representativeness of the cases**	**Selection of controls**	**Definition of controls**	**Most important factor**	**Any additional factor**	**Ascertainment of exposure**	**Same method of ascertainment for cases and controls**	**Non-response rate**	
Farup 2016	1		1		1	1	1	1	1	7
Fukui 2020	1	1	1	1	1	1	1	1		8
Halmos 2015	1	1	1	1	1	1	1	1	1	9
Mortensen 1987		1					1	1	1	4
Ringel-Kulka 2015	1	1	1	1	1	1	1	1	1	9
Sagar 2020	1		1		1	1	1	1	1	7
Sun 2021	1	1	1	1	1	1	1	1	1	9
Tana 2010	1	1	1	1	1	1	1	1	1	9
Tian 2020	1	1	1	1	1	1	1	1	1	9
Vernia 1987		1			1		1	1	1	5

Waseem 2023	1	1	1			1	1	1	1	7
Zhang 2019	1	1	1	1	1	1	1	1	1	9

Supplementary Table S4 Results of Egger's test

	**Bias Coef.**	**Bias 95% Conf. Interval**	**Slope Coef.**	**Slope 5% Conf. Interval**	**P > |t|**
Total SCFAs	-0.3976121	-2.895238, 2.100014	0.0738562	-0.5275169, 0.6752294	0.681
Acetate	1.394528	-2.003903, 4.792959	-0.3301604	-1.161407, 0.5010865	0.354
Propionate	-0.9793771	-5.131996, 3.173241	0.5184281	-0.5032637, 1.54012	0.585
Butyrate	-0.6861996	-2.155867, 0.7634681	0.0924434	-0.2675218, 0.4524086	0.287
Iso-butyrate	-6.271296	-16.47342, 3.930824	1.81723	-1.152308, 4.786768	0.118
Valerate	-0.822878	-5.607888, 3.962132	0.1241238	-1.020154, 1.268402	0.622
Iso-valerate	-5.152876	-13.4714, 3.165648	1.478465	-0.9797311, 3.9797311	0.143

Supplementary Table S5 Results of Sensitive Analysis

	Study omitted	Estimate	[95% Conf. Interval]	
Total SCFAs	Farup (2016)	-0.0537573	-0.25909933	0.15158476
	Halmos (2015)	0.00141935	-0.19591708	0.19875577
	Ringel-Kulka (2015)	-0.02297872	-0.24503924	0.19908179
	Sagar (2020)	-0.02880018	-0.23291819	0.17531782
	Sun (2021)	0.001686	-0.24318291	0.24655491
	Zhang (2019)	0.00478731	-0.19785017	0.20742479
Acetate	Farup (2016)	-0.03316492	-0.21108778	0.14475794
	Fukui (2020)	0.06623571	-0.11704095	0.24951237
	Halmos (2015)	0.00102529	-0.17166284	0.17371342
	Ringel-Kulka (2015)	-0.01878201	-0.20726389	0.16969988
	Sagar (2020)	-0.0122573	-0.18939728	0.16488266
	Sun (2021)	0.05373727	-0.14802383	0.25549838
	Tana (2010)	-0.06147144	-0.23948076	0.11653788
	Tian (2020)	-0.01399851	-0.18906529	0.16106826

Butyrate	Farup (2016)	-0.07053811	-0.25016567	0.109089943
	Fukui (2020)	-0.05677766	-0.24205351	0.1284982
	Halmos (2015)	-0.0579998	-0.23211952	0.1161199
	Ringel-Kulka (2015)	-0.09666561	-0.28707302	0.09374181
	Sagar (2020)	-0.09367953	-0.27238676	0.08502769
	Sun (2021)	-0.0734707	-0.27767083	0.13072941
	Tian (2020)	-0.06368819	-0.24028113	0.11290476
	Zhang (2019)	-0.05968299	-0.23742168	0.11805569

continued table

Iso-butyrate	Farup (2016)	-0.29769403	-1.2220086	0.62662053
	Fukui (2020)	-0.31816927	1.2968056	0.66046709
	Halmos (2015)	0.2100735	-0.08796672	0.50811374
	Sagar (2020)	-0.32388538	-1.2096269	0.56185609
Valerate	Farup (2016)	-0.09725261	-0.3236132	0.12910797
	Fukui (2020)	-0.10922406	-0.34730831	0.12886019
	Halmos (2015)	-0.01943739	-0.23485042	0.19597563
	Sagar (2020)	-0.06992291	-0.29455686	0.15471104
	Sun (2021)	0.00018424	-0.28248942	0.28285789
Iso-valerate	Farup (2016)	-0.21922472	-0.83820349	0.39975408
	Fukui (2020)	-0.17798761	-0.85600758	0.50003237
	Halmos (2015)	0.12230266	-0.14616375	0.39076906
	Sagar (2020)	-0.22224794	-0.82904285	0.38454697
	Zhang (2019)	-0.18330897	-0.80001092	0.433393

Supplemental methods. Full electronic search strategy for each database.

1) PubMed search strategy

#1 "SCFA"[All Fields] OR ("fatty acids, volatile"[MeSH Terms] OR ("fatty"[All Fields] AND "acids"[All Fields] AND "volatile"[All Fields]) OR "volatile fatty acids"[All Fields] OR ("short"[All Fields] AND "chain"[All Fields] AND "fatty"[All Fields] AND "acids"[All Fields]) OR "short chain fatty acids"[All Fields]) OR ("acetalization"[All Fields] OR "acetalizations"[All Fields] OR "acetalized"[All Fields] OR "acetals"[MeSH Terms] OR "acetals"[All Fields] OR "acetal"[All Fields] OR "acetates"[MeSH Terms] OR "acetates"[All Fields] OR "acetate"[All Fields] OR "acetic"[All Fields]) OR ("propionates"[MeSH Terms] OR "propionates"[All Fields] OR "propionate"[All Fields] OR "propionic"[All Fields] OR "propionics"[All Fields]) OR ("butyrates"[MeSH Terms] OR "butyrates"[All Fields] OR "butyrate"[All Fields] OR "butyric"[All Fields]) OR ("valerates"[MeSH Terms] OR "valerates"[All Fields] OR "valerate"[All Fields] OR "valeric"[All Fields]) **538776**

#2 "IBS"[All Fields] OR ("irritable bowel syndrome"[MeSH Terms] OR ("irritable"[All Fields] AND "bowel"[All Fields] AND "syndrome"[All Fields]) OR "irritable bowel syndrome"[All Fields]) **27503**

#3 **#1** AND **#2** **570**

#4 **#3** NOT ("meta analysis"[Publication Type] OR "review"[Publication Type] OR "systematic review"[Filter]) **446**

#5 **#4** AND ((fft[Filter]) AND ((humans[Filter]) AND (english[Filter]))) **181**

2) Web of Science search strategy

#1 TS=(SCFA) OR TS=(SCFAs) OR TS=(short chain fatty acids) OR TS=(volatile fatty acids) OR TS=(acetate) OR TS=(acetic acid) OR TS=(propionate) OR TS=(propionic acid) OR TS=(propanoic acid) OR TS=(butyrate) OR TS=(butyric acid) OR TS=(butanoic acid) OR TS=(isobutyric acid) OR TS=(valerate) OR TS=(valeric acid) OR TS=(pentanoic acid) OR TS=(isovaleric acid) **710325**

#2 TS=(irritable bowel syndrome) OR TS=(IBS) **38867**

#3 **#1** AND **#2** **1093**

#4 **#3** and Review Article or Meeting or Editorial Material or Book or Letter or Case Report (Exclude – Document Types) **600**

#5 **#4** and Humans (MeSH Headings) and English (Languages) **256**

3) Embase search strategy

#1 'short chain fatty acid' OR 'short chain fatty acid'/exp OR 'short chain fatty acids' OR ('short' AND 'chain' AND 'fatty' AND ('acid' OR 'acid'/exp OR 'acids' OR 'acids'/exp)) OR 'scfa' OR 'scfas' OR 'volatile fatty acid' OR 'volatile fatty acids' OR ('volatile' AND 'fatty' AND ('acid' OR 'acid'/exp OR 'acids' OR 'acids'/exp)) OR 'acetate' OR 'acetate'/exp OR 'acetates' OR 'acetates'/exp OR ('acetic' AND ('acid' OR 'acid'/exp OR 'acids' OR 'acids'/exp)) OR 'propionate' OR 'propionate'/exp OR 'propionates' OR 'propionates'/exp OR ('propionic' AND ('acid' OR 'acid'/exp OR 'acids' OR 'acids'/exp)) OR ('propanoic' AND ('acid' OR 'acid'/exp OR 'acids' OR 'acids'/exp)) OR 'butyrate' OR 'butyrate'/exp OR 'butyrates' OR 'butyrates'/exp OR ('butyric' AND ('acid' OR 'acid'/exp OR 'acids' OR 'acids'/exp)) OR 'isobutyrate' OR 'isobutyrate'/exp OR 'isobutyrates' OR 'isobutyrates'/exp OR ('isobutyric' AND ('acid' OR 'acid'/exp OR 'acids' OR 'acids'/exp)) OR ('butanoic' AND ('acid' OR 'acid'/exp OR 'acids' OR 'acids'/exp)) OR 'valerate' OR 'valerate'/exp OR 'valerates' OR 'valerates'/exp OR (('valeric' OR 'valeric'/exp) AND ('acid' OR 'acid'/exp OR 'acids'

OR 'acids'/exp)) OR 'isovalerate' OR 'isovalerate'/exp OR 'isovalerates' OR 'isovalerates'/exp OR ('isovaleric' AND ('acid' OR 'acid'/exp OR 'acids' OR 'acids'/exp)) OR ('pentanoic' AND ('acid' OR 'acid'/exp OR 'acids' OR 'acids'/exp))
596686

#2 'irritable bowel syndrome' OR 'irritable bowel syndrome'/exp OR ('irritable' AND ('bowel' OR 'bowel'/exp) AND 'syndrome') OR 'ibs' **47877**

#3 **#1** AND **#3** **1301**

#4 **#3** AND 'article'/it **601**

#5 **#4** AND 'human'/de AND [adult]/lim AND [english]/lim **164**

181+256+164 = 601 records in total.
